# Simulation in Neurosurgical Education During the COVID-19 Pandemic and Beyond

**DOI:** 10.1017/cjn.2020.234

**Published:** 2021-03

**Authors:** Faizal A. Haji

**Affiliations:** Division of Neurosurgery, Department of Surgery, Faculty of Health Sciences, Queen’s University and Kingston Health Sciences Centre, Kingston, Ontario, Canada

**Keywords:** Simulation, Postgraduate education, COVID-19, Neurosurgery

The COVID-19 pandemic is challenging nearly every aspect of our professional lives, including how we train residents to become competent surgeons. Public health measures instituted to reduce disease spread and safeguard the healthcare system – including postponing elective surgeries, barring learners from the operating room (OR), suspending elective rotations, and shifting to virtual platforms for outpatient care and teaching – have strained our postgraduate education system. These measures have reduced residents’ weekly operative volume by over 50% in some surgical specialties.^1^ Thus, it is not surprising that calls to re-examine the current postgraduate training paradigm have emerged.^2,3^

Simulation-based training (SBT) has been proposed as an alternative to facilitate residents’ ongoing surgical skill development where hands-on training in the OR is not feasible.^3,4^ The paradigm shift toward SBT in surgery began years ago, in response to reduced training opportunities resulting from duty-hour restrictions, concerns for patient safety, and increasing surgical subspecialization.^5^ The rationale for SBT is compelling: simulation provides a “risk-free” environment for trainees to develop surgical skills at their own pace and learn from mistakes without putting patients at risk. In some specialties, the data are similarly compelling: residents whose training is augmented with simulation demonstrate significant improvement in surgical skills both on the simulator and on transfer to the OR, resulting in fewer errors and improved patient outcomes.^6,7^

In this issue, Mirchi et al. advocate for high-fidelity, virtual reality (VR) simulation augmented by intelligent tutoring systems (ITS) to close the training gap created by COVID-19.^3^ The automated feedback generated through ITS reduces the need for a live instructor, increasing the flexibility and feasibility of SBT. Machine learning facilitates individualization of this feedback (particularly in reference to a pre-defined proficiency criterion), which educators could use to adapt simulations “on the fly” based on identified learning needs. Objective data generated by these systems could even provide assessment information that supports existing competency-based medical education curricula. As such, Mirchi et al.’s vision is commendable and has many advantages, particularly if these platforms are developed in accordance with the science of learning and current evidence in simulation instructional design.^8,9^ This literature demonstrates that learning is optimized when trainees engage in repetitive and deliberate practice, interspersed with feedback, distributed over time, on a range of training scenarios varying in difficulty, until mastery is achieved.^9^

Acknowledging these potential advantages, a number of caveats to SBT should be considered. First, VR-based high-fidelity simulation (HFS) may not be appropriate in all circumstances. Despite a pervasive belief that higher fidelity (i.e. more lifelike) simulation leads to better learning, current evidence does not support this notion.^10^ Instead, it is likely that fidelity requirements vary based on learners’ level of expertise and training goals. For novices (e.g. junior residents), low-fidelity simulation produces equivalent learning outcomes to its higher fidelity (and more expensive) counterpart – likely because the latter places excessive information processing demands on inexperienced learners.^11^ Conversely, the potential benefits of high fidelity may be realized among experienced participants (e.g. senior residents and attending surgeons), who can handle these complex environments and may demand a higher level of realism in order to engage with a simulation experience.^11,12^ In addition, high tech does not equate to high fidelity. A high-tech VR simulator that does not provide haptic feedback or realistic tool handling is by definition less realistic (and lower fidelity) than a low-tech bench model that uses real instruments and provides realistic visual and tactile feedback.^13^ The critical issue is not the technology, but rather the alignment between how a surgical skill is practiced on a simulator and how it is subsequently performed in the real world.^14^

Second, the feedback generated from ITS focuses on technical performance (e.g. speed, accuracy, and applied force). While such information is helpful, additional nontechnical skills (e.g. intraoperative communication or situational awareness) and surgical decision-making are not captured. These skills are essential to effective and safe surgical care and should not be ignored.^15,16^ Furthermore, it is not yet known whether residents who receive ITS-derived feedback demonstrate improved surgical performance overall, or simply an improvement in the metrics measured. As such, it is essential that validity evidence for the feedback and/or assessment data generated from these simulation systems are rigorously collected. In particular, we need data regarding changes in the surgical skill of residents receiving ITS-generated feedback, relative to those receiving expert-derived feedback on their performance.^15,16^

The final caveat is that simulation (particularly HFS) is expensive. While subsequent improvements in residents’ skills, surgical errors, and patient outcomes may justify the cost, data demonstrating return-on-investment will likely be necessary before funding is secured. To support this, future studies should report the upfront and recurring costs of simulation platforms. In addition, we need research that investigates whether training on a neurosurgical simulator leads to improved outcomes in the OR.^17^ Notably, while there are over 100 neurosurgical simulation models currently in circulation, only a handful have associated data demonstrating improved performance beyond the simulation environment itself.^15,16^ Future studies will also be more informative if they assess the comparative effectiveness of competing simulation training curricula or compare simulation against other training methods. Such studies help to clarify *how* SBT should be designed, rather than simply justifying that “it works”.^9^

Simulation cannot replace clinical training. The gradually increasing entrustment of residents to perform surgical tasks in the OR as their skills mature should remain the backbone of our training paradigm in neurosurgery. The question that remains is “how and where should simulation be deployed to be of greatest benefit?” In addition to the dedicated learning centers described by Mirchi et al., I believe there is a role for low-tech SBT for junior residents on transferrable skills that are foundational to our specialty (e.g. instrument handling, hemostasis, arachnoid dissection, or suturing under a microscope). Automating such skills in the lab can turn junior trainees into “pre-trained novices”,^17^ who can invest their cognitive resources into higher order skills while in the OR (e.g. identifying and dissecting along a brain–tumor interface). Part-task simulators are likely adequate for this purpose and have already been used in introductory courses like the Canadian Neurosurgery Rookie Camp.^18^ With the ubiquity of 3D printing, some surgical programs are also using portable benchtop simulators so residents can practice basic skills at home during the pandemic.^4^

In addition, simulation could have a substantive impact on improving team function. As technology like the OR Black Box becomes widely available,^19^ our ability to understand surgical errors and latent safety threats improves. Team-based simulation provides a powerful method to address the human factors and root causes of these errors, while also addressing the technical, nontechnical, and team skills necessary to improve surgical care. Conducting such training within authentic clinical contexts (e.g. through insitu simulations in the OR) could further improve surgical outcomes.^20^ Taking this broader perspective will allow the simulation to find its appropriate place within neurosurgical education, during the COVID-19 pandemic and beyond.
